# Mathematical simulation of temperature distribution in tumor tissue and surrounding healthy tissue treated by laser combined with indocyanine green

**DOI:** 10.1186/s12976-019-0107-3

**Published:** 2019-08-19

**Authors:** Yuanyuan Xu, Shan Long, Yunning Yang, Feifan Zhou, Ning Dong, Kesong Yan, Bo Wang, Yachao Zeng, Nan Du, Xiaosong Li, Wei R. Chen

**Affiliations:** 1 Jinzhou Medical University, Jinzhou, 121000 China; 2 Department of Oncology, Graduate Training Base- Fourth Medical Center of Chinese PLA General Hospital of Jinzhou Medical University, Beijing, 100048 China; 3 School of Medicine, Nankai University, Tianjin, 300071 China; 4 Shenzhen University, Shenzhen, 518000 China; 5 Burns Institute, Fourth Medical Center of Chinese PLA General Hospital, Beijing, 100048 China; 6 Department of laboratory animal, Fourth Medical Center of Chinese PLA General Hospital, Beijing, 100048 China; 7 Department of Oncology, Fourth Medical Center of Chinese PLA General Hospital, Beijing, 100048 China; 8 Dalian Institute of Chemical Physics, Chinese Academy of Science, Dalian, 116000 Liaoning China; 9 Biophotonics Research Laboratory, Center for Interdisciplinary Biomedical Education and Research, College of Mathematics and Science, University of Central Oklahoma, Edmond, 73034 USA

**Keywords:** Photothermal therapy, Temperature distribution, Indocyanine green, Pennes bio-equation, Monte Carlo, COMSOL Multiphysics

## Abstract

**Background:**

Photothermal therapy is a local treatment method for cancer and the heat energy generated from it could destroy the tumor cells. This study is aimed to investigate the temperature distribution in tumor tissue and surrounding health tissue of tumor bearing mice applying mathematical simulation model. Tumor bearing mice treated by laser combined with or without indocyanine green. Monte Carlo method and the Pennes bio-heat equation were used to calculate the light distribution and heat energy. COMSOL Multiphysic was adopted to construct three dimensional temperature distribution model.

**Results:**

This study revealed that the data calculated by simulation model is in good agreement with the surface temperature monitored by infrared thermometer. Effected by the optical parameters and boundary conditions of tissue, the highest temperature of tissue treated by laser combined with indocyanine green was about 65 °C which located in tumor tissue and the highest temperature of tissue treated by laser was about 43 °C which located under the tumor tissue. The temperature difference was about 20 °C. Temperature distribution in tissue was not uniform. The temperature difference in different parts of tumor tissue raised up to 15 °C. The temperature of tumor tissue treated by laser combined with indocyanine green was about 20 °C higher than that of the surrounding healthy tissue.

**Conclusions:**

Reasonably good matching between the calculated temperature and the measured temperature was achieved, thus demonstrated great utility of our modeling method and approaches for deepening understand in the temperature distribution in tumor tissue and surrounding healthy tissue during the laser combined with photosensitizer. The simulation model could provide guidance and reference function for the effect of photothermal therapy.

**Electronic supplementary material:**

The online version of this article (10.1186/s12976-019-0107-3) contains supplementary material, which is available to authorized users.

## Background

Photothermal therapy is a local treatment method for cancer which applies intensive laser energy to targeted tumor cells. Heat energy generated from absorbing laser energy could destroy the tumor cells [1]. Photosensitizer such as indocyanine green (ICG) could enhance the absorption of laser energy when it was used in conjunction with laser [2]. The absorption spectrum of ICG is about 600 to 900 nm [3]. ICG irradiated by near-infrared laser could produce thermal effect which shows a severe cytotoxic effect to tumor cells [4]. Many literatures investigated that thermal effect induced by near-infrared laser combined with ICG eradicated the local tumor cells and prolonged the survival time of mice [5, 6]. A clinical trial demonstrated that the thermal effect induced by laser and ICG combined with immunoadjuvant could effectively treated the breast tumor and the side effect was tolerant [7].

Photothermal therapy is an ideal method for cancer treatment which could destroy the targeted tumor cells while protect the surrounding normal tissue. The thermal distribution in tumor tissue and surrounding healthy tissue is the most important factor to influence the effectiveness of photothermal therapy. A literature showed that different biological effect could be induced by different temperatures [8]. For example, when temperature was about 37 °C, the feeling of warmth was felt. When temperature ranged from 60 to 100 °C, the protein could be denatured. When the temperature ranged between 100 °C to 300 °C, the bio-tissue may even be carbonized. In general, tumor cells are sensitive to hyperthermia and vulnerable to heat stress than healthy cells when the temperature was above 42.5 °C [9, 10].

With the development of infrared thermography [11], the digital infrared thermometer can be a reliable method to monitor the surface temperature on tumor. To measure the temperature of deep tissue, thermocouples are always inserted to tissue. However, this method is invasive. During the photothermal therapy, photons coming from laser experience either scatting or absorption when they go through tissue. The extent of scatting and absorption is related to the scatting coefficient and absorption coefficient of tissue respectively. The absorbed photons get excited electronically and in excited state. When transiting from excited state to lower energy state, phones emit energy in some forms, for example, heat generation [12]. The light distribution and temperature distribution during photothermal therapy could be investigated by mathematical simulation, which could display the three dimensional temperature profile of whole tissue not just surface temperature of tissue. Besides, mathematical simulation is a noninvasive method to analyze temperature distribution.

Manuchehrabadi et al. [13] applied the computational Monte Carlo simulation algorithm to simulate the temperature elevation in prostatic tumor embedded in a mouse body during the treatment of laser combined with gold nanorods. In Ganguly’s study [14], finite element modeling was used to demonstrate the temperature distribution and heat affected zone of excised rat skin samples and live anesthetized mouse tissue during laser irradiation. In Paul’s study [15], finite element-based commercial software was used to simulate the subsurface thermal behavior of tissue phantom embedded with large blood vessels during plasmonic photo-thermal therapy. In Sazgarnia’s study [16], the thermal distribution of tumor and surrounding tissue was simulated in COMSOL software in a phantom made of agarose and intralipid during the treatment of laser combined with gold/gold sulfide nanoshells. In Gnyawali’s study [12], finite difference method for heat distribution in tissue was used to simulate the temperature distribution in tissue phantom during the selective laser photothermal interaction. To our knowledge, there was few investigation of simulation model of temperature distribution in tissue phantom during photothermal therapy. The investigations of temperature distribution in living tissue are less. This paper will to investigate mathematics simulation of temperature distribution in tumor tissue and surrounding healthy tissue treated by laser combined with indocyanine green. This study could provide reference function for mathematical simulation design of temperature distribution in tumor and surrounding healthy tissue and provide guidance for the clinical application of photothermal therapy.

## Material and method

### Tumor cell line

4 T1 Cells, a breast tumor cell line, were cultured in Roswell Park Memorial Institute 1640 (RPMI-1640) medium (Invitrogen, Carlsbad, CA) with 10% fetal bovine serum, 100 U/ml penicillin, and 100 U/ml streptomycin (Sigma, St. Louis, MO) at 37 °C in a humidified atmosphere of 5% CO_2_/95% air. The cells were harvested and prepared in the medium (1 million cells per 100 μl) for injection.

### Animal model

Female Balb/c mice (Harlan Sprogue Dawley Co. Indianapolis, IN, USA) at the age of 6 to 8 weeks and weight of 15–25 g were used in our experiment. Mice were anesthetized with a gas mixture of isoflurane (2%) and oxygen before laser irradiation. After the completion of laser irradiation, mice were allowed to recover. All animal experiments were approved by the Institutional Animal Care and Use Committee and were in compliance with National Institutes of Health guidelines. All Balb/c mice were depilated on the back; they were then injected subcutaneously with 10^6^ 4 T1 cells suspended in 100 μl of phosphate-buffered saline. Tumors grew predictably in all mice and reached a size of 5 to 10 mm in diameter 8 to 10 days after injection. Tumor growth was assessed 2 times a week throughout the entire experiment. The orthogonal tumor dimensions (a and b) were measured with a Vernier caliper. The tumor volume was calculated according to the formula, V = ab^2^/2. The tumor-bearing mice were readying for the treatment when the tumor reached 0.2–0.5 cm^3^. Mice were monitored carefully throughout the study and were preemptively euthanized when they became moribund.

### Experimental group

According to the parameters of elements in the photothermal therapy, the experiment was divided into three groups as shown in Table 1. In group 1 and group 3, The tumors were injected with 200 μL of ICG, respectively, the laser power densities were 1 W/cm^2^ and 0.8 W/cm^2^. While in group 2, 200 of μL PBS (Phosphate-buffered saline) was used, and the laser power densities were 1 W/cm^2^.

**Table 1:** The experimental group

Group	Laser power density (W/cm^2^)	The concentration of ICG (mg/ml)
1	1.0	0.1
2	1.0	0.0
3	0.8	0.1

### Photothermal therapy

Before the laser treatment, the 4 T1 tumor-bearing mice were anesthetized, and the hairs overlying the tumor were clipped. Before laser irradiation, 200 μL of ICG solution (Akorn Inc. Buffalo Grove, IL) or PBS was injected into the center of tumors on the back of mice. Eight hundred five nm laser was adopted to irradiate the tumor tissue for 600 s. Infrared thermometer (FLIR E8) was used to measure surface temperature at the irradiation time points of 0, 20 s, 40 s, 60 s, 120 s, 180 s, 240 s, 300 s, 360 s, 420 s, 480 s, 540 s and 600 s.

### Method of temperature distribution simulation model

**Fig. 1:**
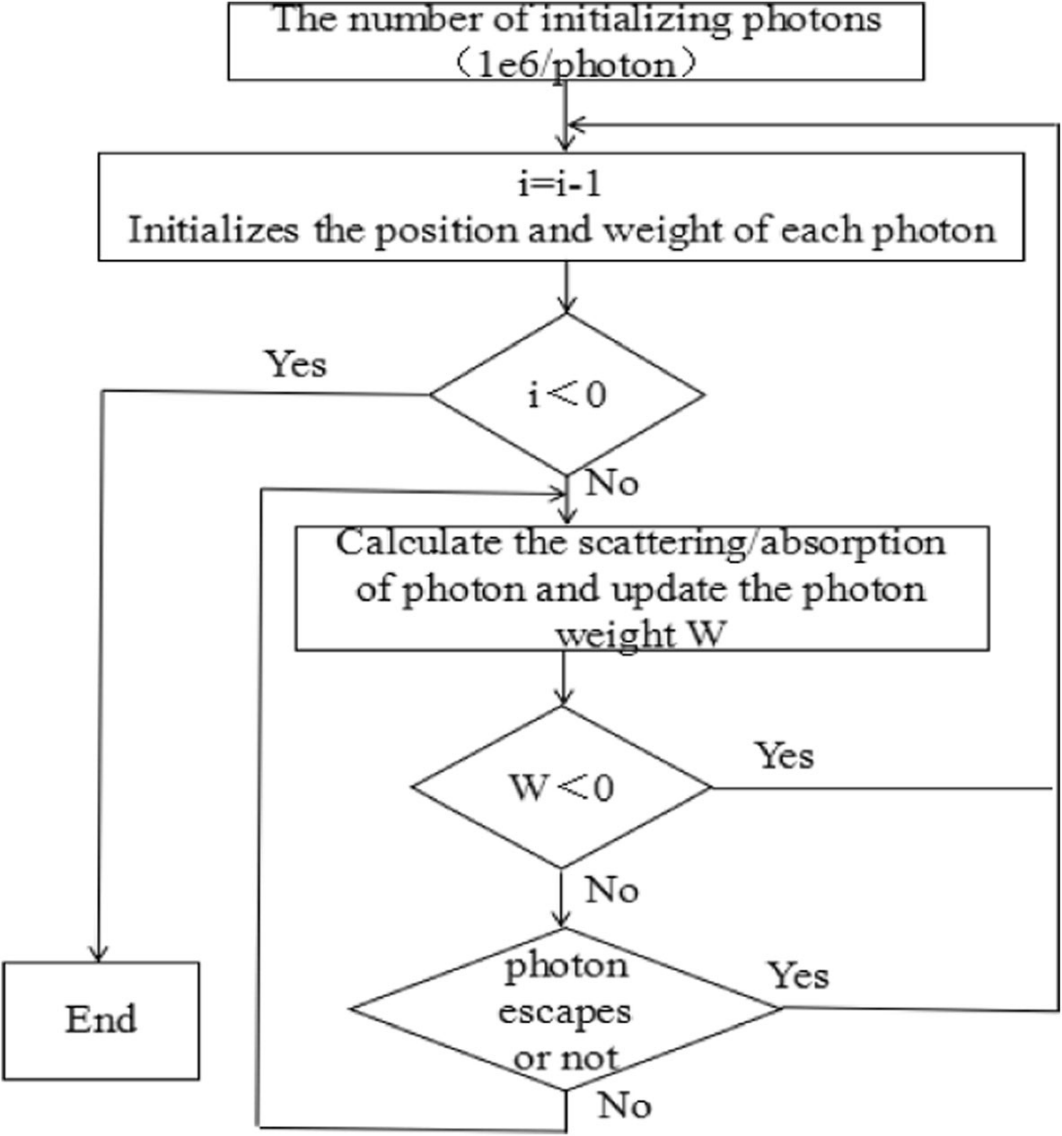
The steps of Monte Carlo simulating light distribution

Monte Carlo methods rely on random sampling to calculate their results which could simulate physical and mathematical systems [17]. Monte Carlo model was capable to simulate the light transportation in multi-layered tissues [18]. The steps of Monte Carlo simulating light distribution were showed in Fig. 1.

**Fig. 2:**
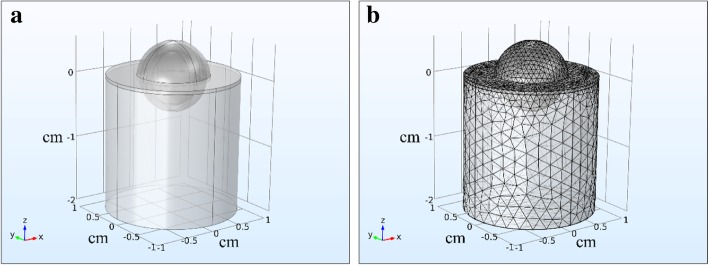
The simulation model of tumor area in the tumor bearing mice. **a**) Diagram of the cylindrical modeling domain of the tumor issue. **b**) A free tetrahedral mesh of the computation domain

Based on the model of breast tumor bearing mice, the physiology of breast tumor area in tumor bearing mice was presented. The breast tumor model was composed of three parts representing skin, fat and tumor. In the simulation model, the thickness of the epidermis and fat above tumor tissue was 0.5 mm and 1 mm respectively. A sphere with a diameter of 8 mm represented tumor tissue and a cylinder with a diameter of 2 cm and height of 2 cm represented the surrounding healthy tissue. The sphere tissue was embedded into the cylinder tissue. The simulated model was showed in Fig. 2.

The model simulated the distribution of the absorption energy which came from an 805 nm laser with a diameter of 1.5 cm. The optical parameters of the tissue [19] were showed in Table 2.

**Table 2:** Optical parameters of tissue

	Absorption Coefficient (μ_a_/cm^−1^)	Scattering coefficient (μ_s_/cm^−1^)	Anisotropy (g)	Refraction index (n)
Skin	0.190	8.340	0.775	1.370
Breast Cancer	0.060	12.75	0.775	1.370
Fat	0.065	10.000	0.775	1.370

In addition of the light energy distribution affected by biological tissue, ICG also contributed a lot to the absorption of light energy. According to the literature study [20], there was a liner relationship about absorption coefficient between ICG and 805 nm laser as follows:

1 $$ \mathrm{A}=0.04\cdot {\mathrm{C}}_{\mathrm{ICG}} $$

A is the absorption coefficient of ICG under the irradiation of 805 nm laser. C_ICG_ (μg/mL) is the concentration of ICG. When tumor tissue was treated by laser combined with photosensitizer, the absorption coefficient was equal to the sum of the light absorption coefficient of tumor tissue and the light absorption coefficient of the photosensitizer.

Heat distribution of tissues was calculated by Pennes bio-heat equation. The Pennes bio-heat equation reads:

2 $$ \uprho \mathrm{C}\frac{\mathrm{\partial T}}{\mathrm{\partial t}}-\nabla \left(\mathrm{k}\cdot \nabla \mathrm{T}\right)={\uprho}_{\mathrm{b}}\cdot {\mathrm{C}}_{\mathrm{b}}\cdot {\upomega}_{\mathrm{b}}\cdot \left({\mathrm{T}}_{\mathrm{b}}-\mathrm{T}\right)+{\mathrm{Q}}_{\mathrm{met}}+{\mathrm{Q}}_{\mathrm{ext}} $$

where ρ (kg/cm^3^), C (J/((kg∙K)))and k are the density, specific heat and thermal conductivity of the tissue respectively. T is the temperature, ω_b_ (1/s), ρ_b_ (kg/cm^3^), C_b_ (J/((kg∙K)))and T_b_ (C) are the perfusion, density, specific heat and the temperature of the blood, Q_met_ (W/m^3^) is the metabolic heat generation rate per unit volume of the tissue, Q_ext_ (W/m^3^) is the distributed volumetric heat source due to laser heating. The data of Q_ext_ came from Monte Carlo simulation which calculated the energy of light distribution in tissues. The temperature distribution simulation of tissues during the photothermal therapy was performed via the finite element method available in COMSOL Multiphysics computational package. Thermophysical simulation was consist with the model of light distribution. A set of thermophysical parameters of tissues were used in the simulation as shown in Table 3.

**Table 3:** Thermal parameters of tissue [21–24]

	ρ (kg/m^3^)	C (J/((kg∙K)))	k (W/(m∙K))	ω_b_ 1/s	Q_met_ (W/m^3^)
Skin	1180	2291	0.58	0.0005	420
Breast Cancer	1150	4200	0.561	0.0036	420
Fat	1000	3148	0.58	0.0005	420

The boundary of the epidermis in the simulation was the boundary of air convection, and the convective heat transfer coefficient was 18(W/m^2^ ∙ K). The environment temperature was selected at 15 °C and considered constant. Other boundaries temperature was 37 °C.

## Results

### Surface temperature distribution during laser irradiation

**Fig. 3:**
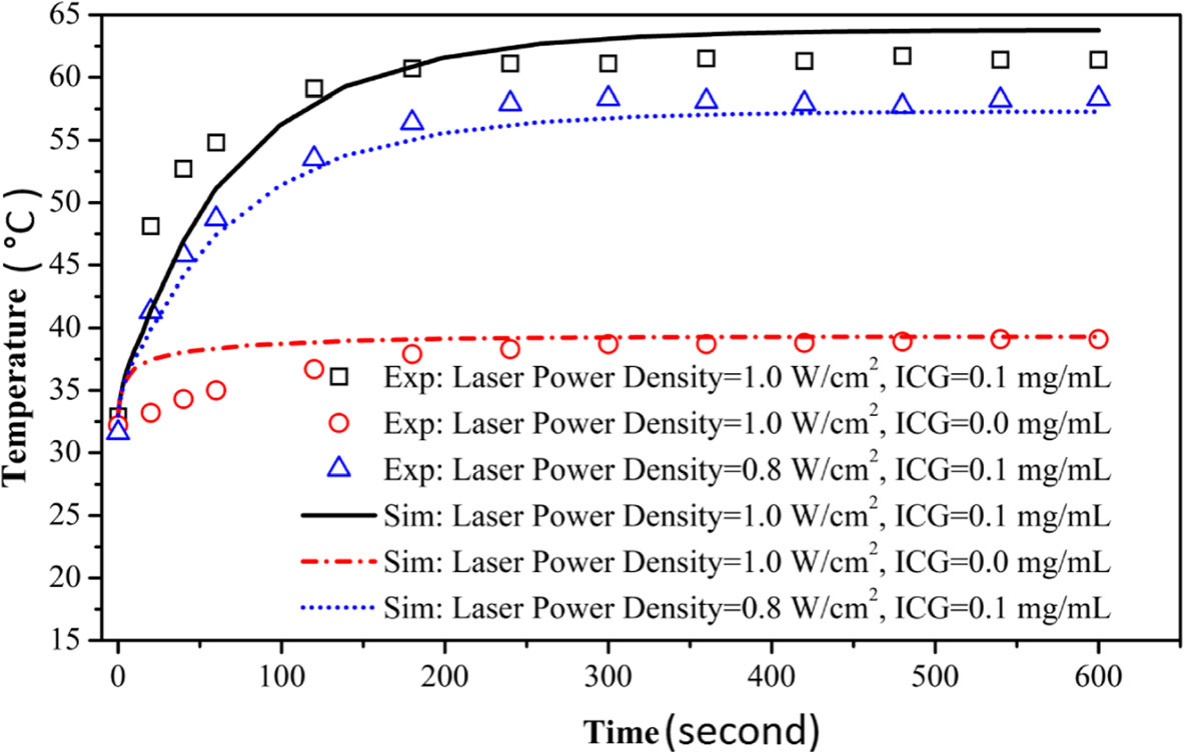
Comparison of the experimental and simulated results on the surface tumor temperature in tumor bearing mice

The surface temperature of tumor tissue was monitored by infrared thermometer and calculated by simulation model, as shown in Fig. 3. In the first 240 s of photothermal therapy, the temperature rose rapidly, then the temperature was not obviously elevated and became stable after 240 s. The temperature of tumor in group 1 (solid line - square) and group 2 (dash dot line - circular) were about 63 °C and about 39 °C respectively at t = 600 s. The maximum temperature difference was about 20 °C between the two groups. The results showed that ICG contributed a lot to temperature elevation. The temperature difference between group 1(solid line - square) and group 3 (short line - triangle) was about 5 °C. The temperature measured in experiment was almost consistent with the temperature calculated by the simulation, especially after 240 s.

### Monte Carlo simulation of light distribution in tissues

**Fig. 4:**
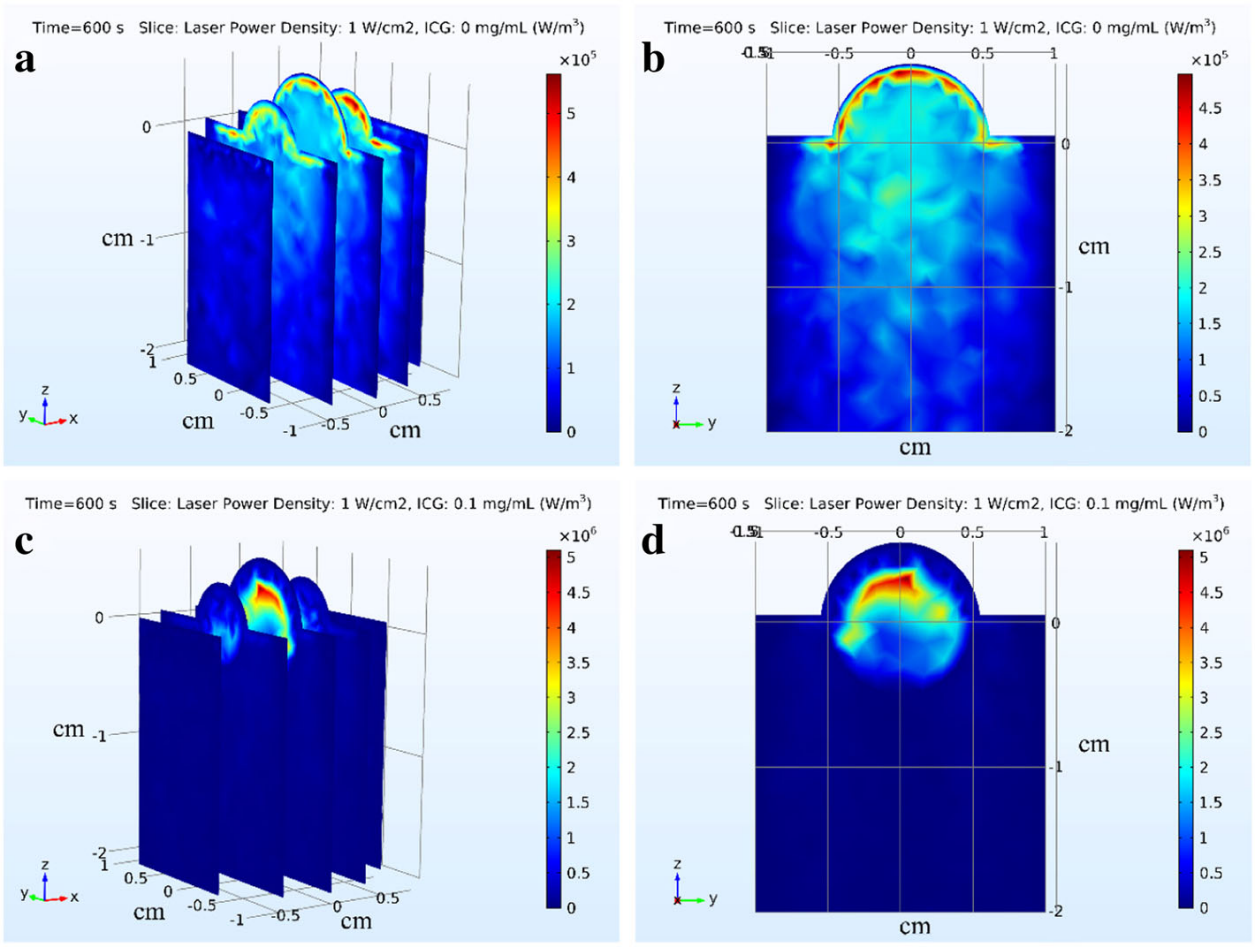
The distribution of the absorbed laser energy (W/m^3^) in tumor and surrounding tissue. **a**, **b** The laser power density is 1 W/cm^2^ and the ICG is 0.0 mg/mL. **c**, **d** The laser power density is 1 W/cm^2^ and the ICG is 0.1 mg/mL

The light distribution in tumor tissue and surrounding healthy tissue was simulated by Monte Carlo method, as shown in Fig. 4. When tumor was irradiated by laser (Fig. 4a and b), the light energy absorbed by tumor tissue was almost equal to that absorbed by surrounding healthy tissue. The area had the maximum absorption light energy locating in the tumor tissue where it was about 1.5–2 mm from the epidermis. The maximum absorption energy was 5 × 10^5^ W/m^3^.

When the tumor had been injected with ICG and irradiated by laser (Fig. 4c and d), the dose of light energy absorbed by tumor tissue was more than that absorbed by surrounding healthy tissue. The largest absorption of light energy in tumor tissue and surrounding healthy tissue were 5 × 10^6^ W/m^3^ and 0.5 × 10^6^ W/m^3^ respectively. The area had the maximum absorption light energy locating in the tumor tissue where it was about 5–7 mm from the epidermis.

### Temperature distribution in tissue at different treatment parameters

**Fig. 5:**
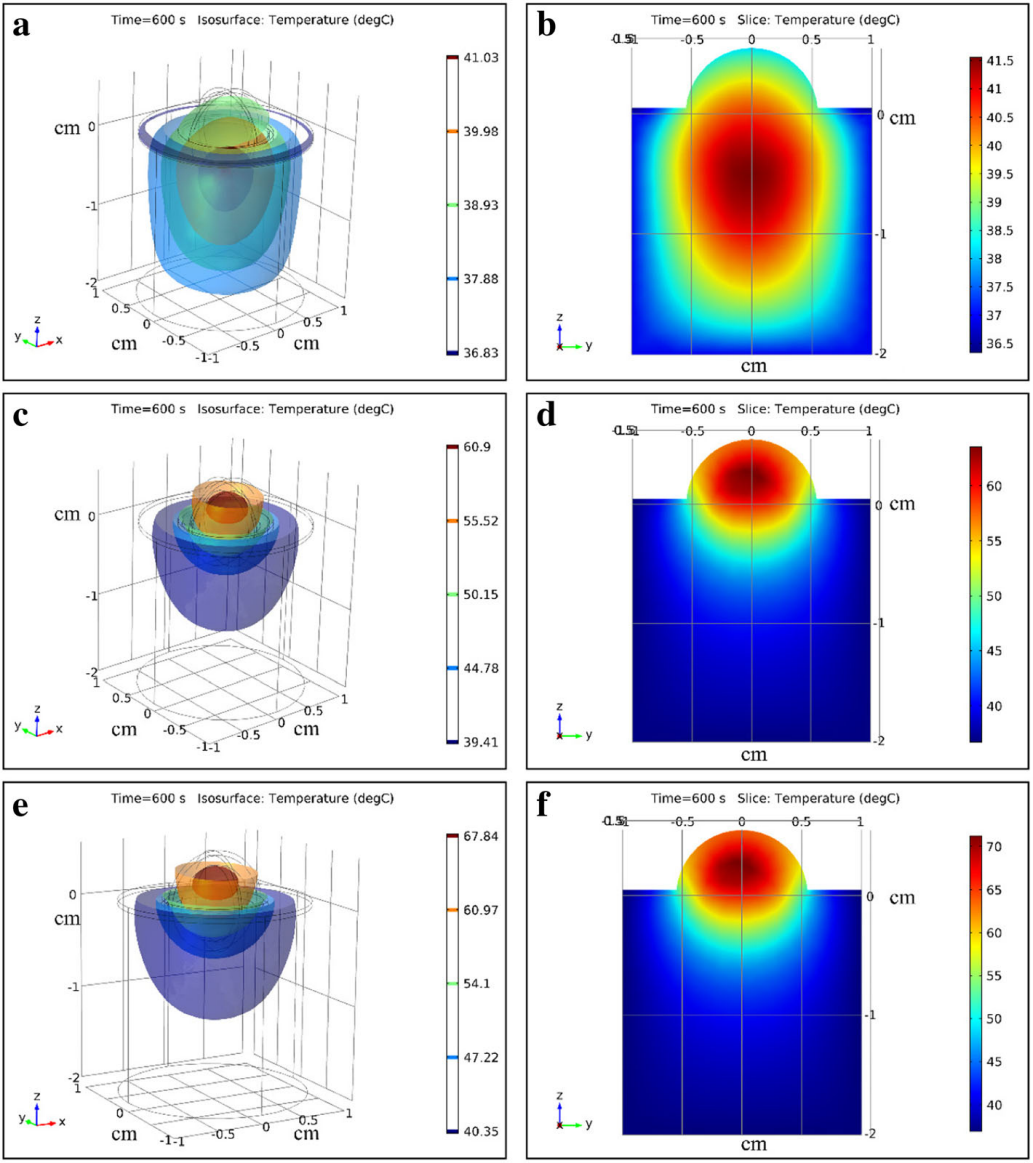
Three-dimensional and two-dimensional temperature distributions in tumor tissue and surrounding healthy tissue during photothermal therapy. **a**, **b** The laser power density is 1 W/cm^2^ and the ICG is 0.0 mg/ml. **c**, **d** The laser power density is 0.8 W/cm^2^ and the ICG is 0.1 mg/mL. **e**, **f** The laser power density is 1 W/cm^2^ and the ICG is 0.1 mg/mL


When tissue was irradiated for 600 s, the temperature distribution of tumor tissue and surrounding healthy tissue at different treatment parameters was showed in Fig. 5 (Additional file 2). When tumor bearing mice were treated by laser combined with ICG (Fig. 5c, d, e and f), temperature of tumor tissue was significantly higher than the surrounding healthy tissue. The highest temperature at t = 600 s (Fig. 5e and f) in tumor tissue and surrounding healthy tissue were about 70 °C and 50 °C respectively when tumor was treated by laser (1 W/cm^2^) and ICG (0.1 mg/ml). The position had the highest temperature locating in the tumor tissue where it was about 5–8 mm from the epidermis. The surface temperature of tumor tissue was about 65 °C. The temperature difference between the highest temperature and the lowest temperature in tumor tissue was about 20 °C in Fig. 5e, f and 15 °C in Fig. 5c, d.

**Additional file 1:** Temperature Evolution in tumor and surrounding tissue by laser without ICG (2). (AVI 3180 kb)


Temperature distribution was showed in Fig. 5a and b when tumor bearing mice was treated by laser without ICG. The highest temperature was about 41.5 °C under the tumor tissue. The temperature of tumor tissue ranged between 37 °C to 41.5 °C. The temperature of surrounding healthy tissue was about was about 38.5 °C at t = 600 s.

### Temperature distribution during photothermal therapy at different time

**Fig. 6:**
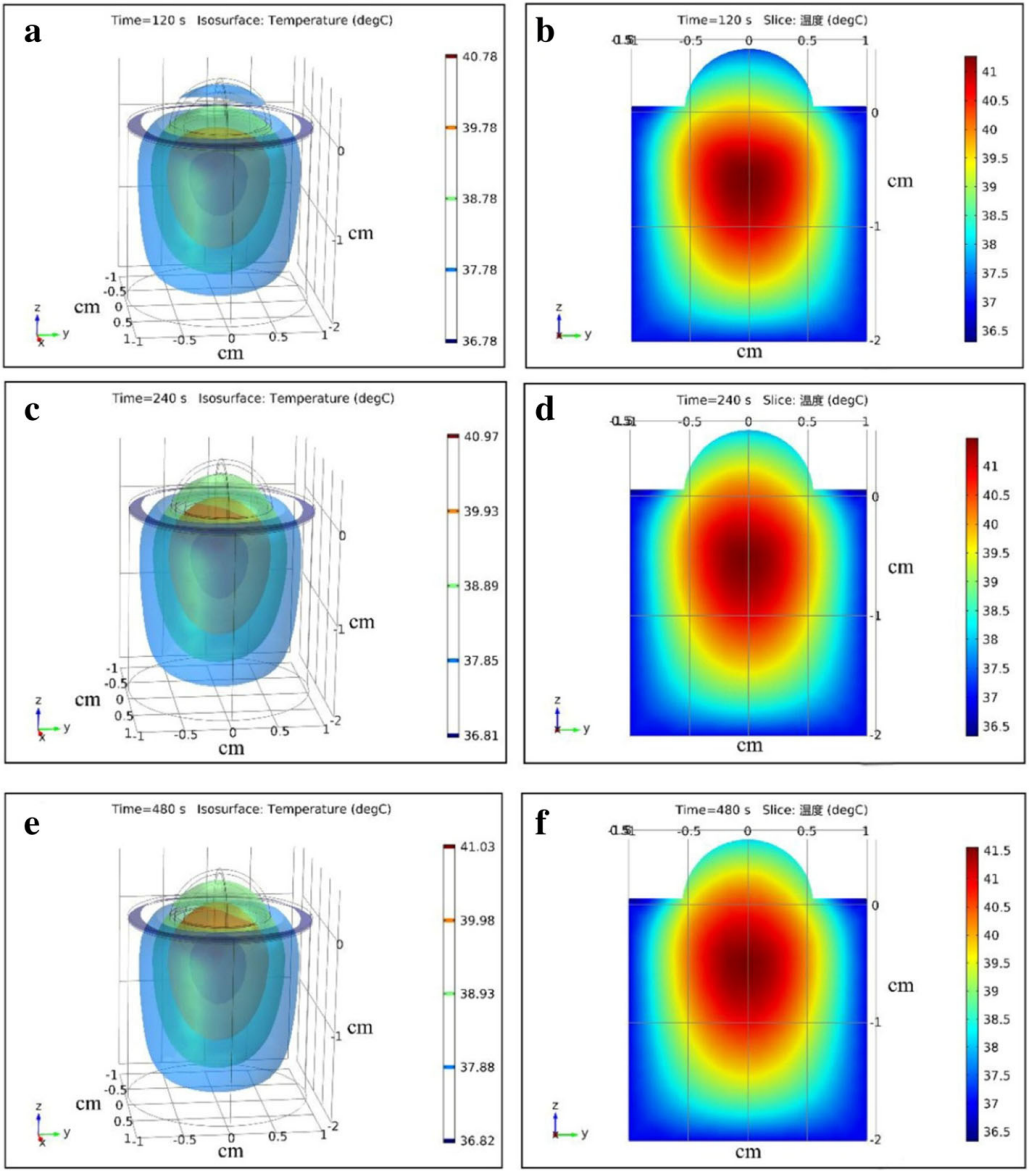
Three-dimensional and two-dimensional temperature distribution in tumor tissue and surrounding tissue treated by laser without ICG. **a**, **b** t = 120 s, **c**, **d** t = 240 s, **e**, **f** t = 480 s


The two-dimensional and three-dimensional temperature distribution of tumor tissue and surrounding healthy tissue treated by laser without ICG at different time were showed in Fig. 6 (Additional file 1). The body temperature of mice was about 37 °C. The area of the highest temperature was under the tumor where it was about 13–18 mm from the epidermis. The highest temperature varied from 37 °C to 41.5 °C. The surface temperature varied from 32 °C to 38.5 °C.

**Additional file 2:** Temperature Evolution in tumor and surrounding tissue by laser with ICG (2). (AVI 3310 kb)


**Fig. 7:**
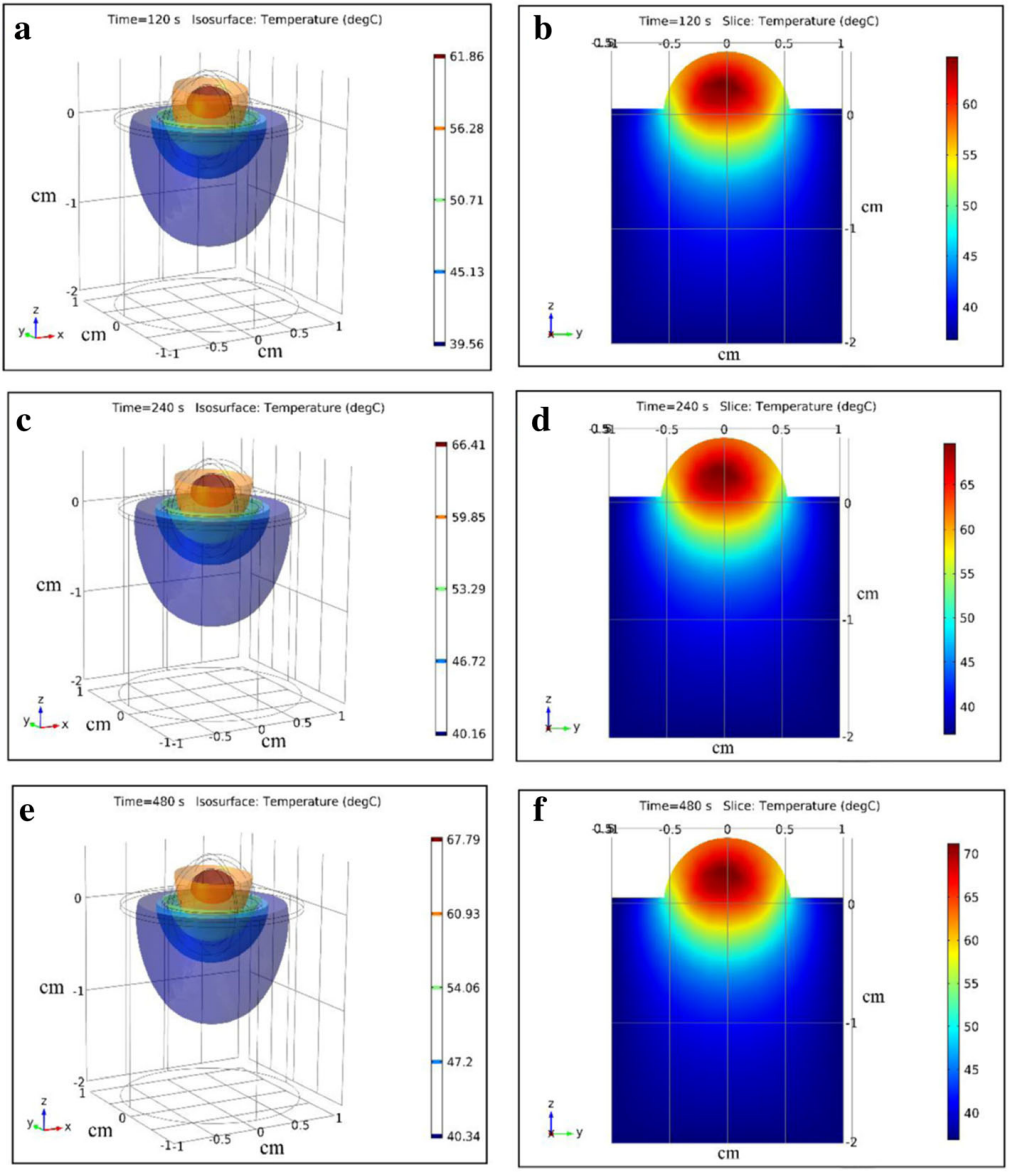
Three-dimensional and two-dimensional temperature distribution in tumor tissue and surrounding tissue treated by laser with ICG. **a**, **b** t = 120 s, **c**, **d** t = 240 s, **e**, **f** t = 480 s

The two-dimensional and three-dimensional temperature distribution of tumor tissue and surrounding healthy tissue treated by laser (1 W/cm^2^) combined with ICG (0.1 mg/ml) at different time were showed in Fig. 7. The area of the highest temperature was in the tumor where it was about 5-8 mm from the epidermis. The highest temperature varied from 37 °C to 70 °C. The maximum temperature of surrounding tissue was about 50 °C.

## Discussion

In this work, temperature distribution of tumor tissue and surrounding healthy tissue was investigated when tumor bearing mice was treated by laser with or without ICG. The infrared thermometer was applied to measure the surface temperature during photothermal therapy. Based on the model of tumor bearing mice treated by photothermal therapy, mathematical simulation about temperature distribution was constructed. The model coupled the physical light field and heat field. According to the generation principle of heat and light field, the constructed simulation model in this study included two parts. Firstly, light distribution in the tumor and surrounding healthy tissue was simulated by Monte Carlo method, and then the energy distribution of heat source was calculated according to light distribution and absorption coefficient of tissue and ICG. Secondly, based on Pennes bio-heat equation, temperature field simulation model of tumor tissue and surrounding healthy tissue was constructed by using direct coupling analysis software COMSOL Multiphysics. The simulated results were compared with the measured results in the vivo experiment. To our knowledge, it is the first work to investigate the temperature distribution of tumor bearing mice treated by laser combined with ICG. Besides, it is the first time to analyze the spatial and temporal temperature simulation model according to the combination of Monte Carlo method and the finite element method available in COMSOL Multiphysics.

The simulation results were in good agreement with the experimental results, as shown in Fig. 3. The present results about temperature distribution of living tissue matched well with the results about tissue phantoms demonstrated by Gnyawali SC. In Gnyawali SC’s study [12], Gelatin phantoms were applied to simulate normal biological tissue. A spherical ICG-mixed gelatin buried in the gelatin was applied to simulate tumor tissue which could simulate absorption-enhanced target for selective photothermal interaction. An 805 nm laser was used to irradiate the dye for 600 s and a Prism DS-infrared camera was used to monitor the real-time surface temperature. The Monte Carlo method and finite difference method were used to simulate the surface temperature profile about the tumor tissue. The simulated results and the experimental results were in good agreement. The current experimental results provided a more valuable role for the clinical application of photothermal therapy compared with the results of tissue phantoms. The result showed that temperature monitoring is feasible using mathematical simulation.

The temperature simulation model contained the coupling of the light field and the heat field. The light distribution was simulated by the Monte Carlo method. Monte Carlo simulation method is a kind of commonly used statistical simulation random sampling method, which has been widely used in the simulation of various random processes. Light distribution of complex organization can be regarded as the results of a large number of photons randomly moving and absorbed in the tissues which could be investigated by Monte Carlo method [25, 26]. Xue Lingling’s research [27] showed that the simulation results of five layer of skin tissue solved by Monte Carlo method fit well with the experimental results. The heat energy distribution was simulated by Pennes bio-heat equation. The Pennes bio-heat equation is a classical bio-heat equation which considered the effect of the blood perfusion, metabolism heat generation of tissues as well as the heat absorption of ICG. Monte Carlo simulation provided the heat energy source for Pennes bio-heat equation. COMSOL Multiphysics is a multi-physical field coupling software which was used to couple the light and heat physical fields. The mathematical simulation model of this study conforms to the heat transfer characteristics of biological tissue which make the simulation results agreed with the experiment results.

Figure 5 showed the light distribution of tumor tissue and surrounding healthy tissue. The absorption energy deposition was affected by the optical parameters of tissue and the absorption coefficient of ICG. The pattern of light energy distribution in tissue was largely due to the concave shape of the tumor top surface where the laser is incident and the cylinder-shaped of surrounding tissue. The light energy distribution was similar to the results showed by Manuchehrabadi [13] who applied the Monte Carlo method to simulate photon propagation in a spherical tumor and calculate laser energy absorption in tumor tissue.

When the tumor tissue was treated by laser without ICG (Fig. 6), the temperature of tumor tissue and surrounding tissue was not above 42.5 °C. The tumor and surrounding healthy tissue would not be damaged by laser. Referring to the optical parameters and boundary conditions of tissue, the simulation showed that the highest point of the temperature field was under the tumor tissue when tumor was not treated by ICG. The highest point of the temperature field was in the tumor tissue and close to the skin when the tumor was deposited with ICG. The temperature distribution was similar to the results reported by Manuchehrabadi N et al. [13].

Mathematical simulation demonstrated that the temperature of the tumor tissue was higher than the temperature of surrounding healthy tissue under the treatment of laser combined with ICG (Fig. 7). Temperature distribution of the tumor was not uniform. The temperature of different part of tumor tissue varied from about 45 °C to 70 °C. In general, temperature of the tumor periphery is lower than the temperature of the central region. As literature mentioned [9, 28], when the temperature of tumor cells was above 42.5 °C, the number of dead tumor cells drastically increased with increasing temperature. The temperature of surrounding healthy tissue varied from 37 °C to about 45 °C. Within this temperature, the surrounding tissue near the tumor tissue could be destroyed slightly and the tissue far away the tumor could be relatively safe.

During photothermal therapy, temperature elevated obviously before t = 240 s. While the temperature become stable after 240 s. The variation trend of temperature was also observed in the Gnyawali’s study [12]. The tumor in group 1 and group 3 had the same concentration of ICG, they were irradiated by laser with power density of 1 W/cm^2^ and 0.8 W/cm^2^ respectively, the maximum temperature difference was about 5 °C. Compared with ICG, the contribution of laser power density to temperature elevation seemed not obvious. Kannadorai et al. [29] also found that there was hardly any increase in overall temperature of the tumor during the photothermal therapy when laser power density was steadily increased. Maybe, the laser power density contributed a little to the temperature elevation.

There are still some drawbacks to this experiment. The geometric structure in this study was fixed and could not simulate the different tumor size, tumor shape and tumor depth which caused tiny inconsistency between simulation results and experiment results. Further studies in this subject will be investigated in the future. In this study, the distribution of ICG was thought to be uniform. However, instability and easy biodegradation are the characteristics of ICG. A literature [30] investigated that graphene oxide-titanium dioxide nanomaterial/ICG (TiO_2_-GO / ICG) was stable and could increase tumor accumulation of ICG when TiO_2_-GO / ICG was used for cancer treatment as a photosensitizer. The temperature distribution of ICG loaded by nanomaterial will be a direction to be investigated.

## Conclusion

Mathematical simulation was feasible to monitor the temperature of tissue during photothermal therapy. The simulation model could predict the temperature distribution in tumor tissue and surrounding healthy tissue to achieve the ideal effectiveness of treatment that could selectively destroy the tumor cells while avoid damaging the surrounding healthy tissue. Photosensitizer, ICG, could selectively elevated the temperature of tumor tissue. The model could provide guidance function for the research and development of appropriated photosensitizer which could targeted to tumor cells and be uniform distribution in tumor tissue. The appropriated photosensitizer should be further researched and developed. The best thermal dose should be further investigated and the model of temperature distribution could provide guidance function.

## Nomenclature

- ρ the density, kg/cm^3^
- C the specific heat, J/((kg∙K)
- k the thermal conductivity, W/(m∙K)
- Q_met_ the metabolic heat generation rate per unit volume of the tissue, W/m^3^
- Q_ext_ the distributed volumetric heat source due to laser heating, W/m^3^
- ρ_b_ the blood density, kg/cm^3^
- C_b_ the blood specific heat, J/((kg∙K)
- ω_b_ the blood perfusion, 1/s
- T_b_ the blood temperature, °C

## Data Availability

All data generated or analyzed during this study are included in this published article and its additional file.

## Abbreviations

ICG: Indocyanine green
PBS: Phosphate-buffered saline
RPMI-1640: Roswell Park Memorial Institute 1640
